# Effects of Transcranial Direct Current Stimulation Over the Left Primary Motor Cortex on Verbal Intelligence

**DOI:** 10.3389/fnhum.2022.888590

**Published:** 2022-05-26

**Authors:** Yifan Huang, Yinling Zhang, Yizhe Zhang, Xiaoqin Mai

**Affiliations:** ^1^Department of Psychology, Renmin University of China, Beijing, China; ^2^Psychological Counseling Center, Shanghai University, Shanghai, China

**Keywords:** verbal intelligence, performance intelligence, left primary motor cortex, transcranial direct current stimulation (tDCS), anodal stimulation

## Abstract

Previous studies have shown that changes in gray matter density and volume in the left primary motor cortex are significantly associated with changes in individuals’ verbal intelligence quotient (VIQ), but not with their performance intelligence quotient (PIQ). In the present study, we examined the effects of transcranial direct current stimulation (tDCS) over the left primary motor cortex on performance in intelligence tests. We chose four subtests (two each for VIQ and PIQ) of the Wechsler Adult Intelligence Scale-Chinese Revised version and randomized participants into anodal, cathodal, and sham groups. We found that anodal stimulation significantly improved performance in verbal intelligence subtests compared to cathodal and sham stimulation, while performance intelligence subtest scores did not change in any stimulation condition. These findings suggest that the excitation level of the left primary motor cortex has a unique effect on verbal intelligence.

## Introduction

Among current theories on the meaning and structure of intelligence, one of the most influential theories is the fluid and crystallized theory of intelligence propounded by Cattell (1941). Fluid intelligence refers to the ability to reason and solve novel problems, such as inductive, deductive, and quantitative reasoning, while crystallized intelligence is described as the ability to use acquired knowledge, experience, and language skills. In the measurement of intelligence quotient (IQ), fluid intelligence is often assessed by perceptual reasoning tasks, corresponding to performance IQ (PIQ), and crystallized intelligence is often tested by lexical tasks, corresponding to verbal IQ (VIQ). Some early studies have shown that these two kinds of intelligence present different developmental patterns across the lifespan, since fluid intelligence peaks in middle adolescence and begins to decline in the mid-twenties, while crystallized intelligence continues to develop during adulthood and is less likely to deteriorate with aging (Cattell, 1963; Wang and Kaufman, 1993; Craik and Bialystok, 2005).

Not only do fluid and crystallized intelligence show differences in measurement and developmental trajectories, evidence from neuroimaging studies suggests that they also have distinctive neural mechanisms. Many studies have examined the structural and functional differences between different types of intelligence. Early exploration by Andreasen et al. (1993) showed that performance intelligence (which corresponds to fluid intelligence) was positively correlated with overall gray matter volume in the brain, while the association between this intelligence and specific brain structures was relatively modest compared to that of full-scale and verbal intelligence. Similar findings were observed in a recent study, where a robust correlation was found between total brain volume (also mainly interpreted by gray matter volume) and fluid intelligence (Nave et al., 2018). In addition, other studies found that performance intelligence were positively predicted by cortical thickness in a wide network of bilateral brain areas (Karama et al., 2011) and the degree of hemispheric volume asymmetry (Raz et al., 1993). In addition, Jung and Haier (2007) proposed the parieto-frontal integration theory of intelligence, postulating a distributed network of regions essential for intellectual activities, which consists of parts of the dorsolateral prefrontal cortex, superior parietal lobule, inferior parietal lobule, anterior cingulate gyrus, temporal lobe, and occipital lobe. The efficiency of the network connectivity and some neuroanatomical indices, such as larger volume, higher density and increased activation of these areas, can be used to explain individual differences in complex visuospatial and reasoning tasks (Choi et al., 2008; Deary et al., 2010; Ritchie et al., 2015). The association between this network and fluid intelligence has been validated in many studies (Langeslag et al., 2013; Genc et al., 2019; Gur et al., 2020).

Compared to the association with functional brain activities shown by performance intelligence, performance on verbal intelligence appears to be more closely related to brain structure (Witelson et al., 2006; Choi et al., 2008). A link between the temporal cortex (especially the anterior temporal cortex) and verbal intelligence has been suggested, as Choi et al. (2008) found that total and sub-dimensional scores on verbal intelligence tests correlated with cortical thickness in different regions of the left temporal lobe. In a recent study using histological analysis and cellular recordings of neurosurgically resected temporal cortex, researchers found that higher verbal intelligence was associated with thicker left (but not right) middle temporal cortex (Heyer et al., 2021). As long-term memory is considered as underlying the storage of semantic concepts, and such a process relies on a distributed network that includes the anterior cingulate, lateral prefrontal, and temporal cortices, the correlation between verbal intelligence scores and the temporal cortex may reflect the application of stored semantic knowledge to such intelligence tasks. In addition, considering that crystallized intelligence is closely related to language ability, some researchers combined voxel-based morphometry and region of interest-based analysis to explore the relationships between verbal intelligence and classical language-related brain regions such as Broca’s area and Wernicke’s area, and found that the cortical thickness of Broca’s area in the left hemisphere was significantly correlated with individuals’ verbal intelligence test scores (Konrad et al., 2012).

As above evidence mostly reflected correlations between the verbal intelligence and certain areas in the left hemisphere, a previous study demonstrated that verbal expression was more closely associated with the left hemisphere (Gazzaniga and Sperry, 1967). In patients with unilateral brain injury, there was a significant association between decreases in verbal intelligence and damage to the left hemisphere (Warrington et al., 1986). Raz et al. (1987) performed computed tomography scans on patients with probable dementia of Alzheimer’s disease and found that the severity of left-hemisphere atrophy correlated with the degree of decline in verbal intelligence. A large sample study of a healthy population, provided further evidence that verbal intelligence was positively correlated with the gray matter volume of wide-ranging areas in the left hemisphere, including the left gyrus rectus and anterior cingulate gyrus, left posterior insula and planum polare, and left superior and middle frontal gyri (Hidese et al., 2020). It can be speculated from the above studies that the structures of the left hemisphere may be more related to the variation in verbal intelligence. Furthermore, verbal intelligence tasks often involve multiple types of cognitive processes, such as the application of acquired semantic knowledge, semantic comprehension, speech production, and so on, indicating that verbal intelligence may relate to extensive areas of the left hemisphere.

Ramsden et al. (2011) showed that the left motor cortex may play an important role in the development of verbal intelligence. In their study, they used the Wechsler Intelligence Scale (Wechsler, 1981) to test the intelligence level of 33 teenagers in 2004 and again in 2007 (or 2008), combined with structural magnetic resonance imaging each time. The researchers found that VIQ changed with the increase of gray matter density in the left motor speech area while PIQ changes were related to gray matter density in the anterior cerebellum, indicating a dissociable structural change with the development of different kinds of intelligence (Ramsden et al., 2011). Ljunggren et al. (2015) used a series of intelligence tests to examine cognitive performance prior to and 2 years after surgery in epilepsy patients who underwent frontal lobe resection. They found that patients who had surgery at the premotor or supplementary motor area showed a reliable decrease in lexical comprehension. These findings indicated that there may be a correlation between the development of verbal intelligence and structural changes in the left motor cortex. However, to date, such evidence still cannot support a casual role of the motor cortex in verbal intelligence.

Transcranial direct current stimulation (tDCS) is a low-cost, safe, and non-invasive brain stimulation technique that has been widely used to improve cognitive and motor functions (Fregni et al., 2015; Riggall et al., 2015). This technique modulates cortical excitability by applying weak electrical current to the target brain region and causes depolarization and hyperpolarization of resting membrane potentials (Stagg and Nitsche, 2011). Meanwhile, the modulating effect of tDCS can be maintained for a period of time after the end of stimulation, for instance, after applying 20 min of 2 mA anodal stimulation to the primary motor cortex, researchers found that the promotion of cortical excitability could last for up to 60 min (Jamil et al., 2017), showing a beneficial effect of tDCS on neuron plasticity. As an effective technique of exploring causal relationships between specific brain regions and cognitive functions, such as language and memory (Hertrich et al., 2016; Branscheidt et al., 2017), tDCS helps further reveal the relationship between the left motor cortex and verbal intelligence.

As mentioned above, crystallized intelligence primarily reflects the ability to utilize stored semantic knowledge and expressive skills, and is usually measured by verbal intelligence tasks such as lexical or verbal analogy tests (Genc et al., 2019). No prior study has tested whether enhancing activation of the left motor cortex by tDCS would improve performance in verbal intelligence. However, given the complexity of the cognitive processes covered in verbal intelligence tests, of which speech production is also an important part, a number of studies that found improvement in articulation tasks by administering tDCS on this region are important for exploring the causal relationship between the left primary motor cortex and verbal intelligence. Liuzzi et al. (2010) examined the effect of tDCS on the acquisition of novel action words, and found that a group receiving cathodal tDCS over the left motor cortex performed poorly in translating novel words into their native language compared to control group receiving sham stimulation. This effect disappeared when the stimulation was applied to the prefrontal cortex, demonstrating the specific role of the motor cortex in vocabulary learning and retrieval. Other studies reported that tDCS applied to the primary motor cortex could improve language function in healthy older adults and individuals with post-stroke aphasia (Meinzer et al., 2014, 2016). One recent study of patients with post-stroke aphasia found that, combined with speech therapy, anodal tDCS administered on the left primary motor cortex (A-tDCS-M1 group) significantly promoted participants’ performance in tasks such as auditory word-picture matching and picture naming, and this positive effect was more evident than that in the group receiving stimulation in Broca’s area and the sham group (Wang et al., 2019). In the same study, results for approximate entropy revealed that not only the left motor cortex but also the dorsolateral prefrontal cortex and Broca’s area in the ipsilateral hemisphere were significantly activated in the A-tDCS-M1 group, suggesting that stimulation over the left motor cortex may result in an extensive effect on the activation of other speech-related areas in the articulatory network. In contrast to the possible correlation between verbal intelligence and the left primary motor cortex revealed by the above findings, little association between performance intelligence and this region has been reported. Summarizing the available evidence, a correlation between activity in prefrontal areas and performance intelligence has been observed (Nisbett et al., 2012), which was also confirmed by a tDCS study (Zmigrod et al., 2014).

In summary, accumulated evidence indicates an association between the left primary motor cortex and verbal intelligence. Nonetheless, currently no studies are available using specific intelligence tests to examine the causal relationship between this area and verbal task performance in a healthy population. Therefore, in the present study, we aimed to use tDCS to investigate whether changes in cortical excitability of the left motor cortex could specifically influence performance in verbal intelligence tasks. We randomized subjects into three group (anodal, cathodal, and sham) and assessed their intelligence before and after stimulation. The test battery used here was the Wechsler Adult Intelligence Scale Revised Chinese version (WAIS-RC), a common tool for intelligence measurement that consists of verbal intelligence and performance intelligence subtests. Based on findings from previous studies and the consideration that tDCS is a polarity-dependent technique, we proposed the following hypotheses. (1) In the verbal intelligence subtests, anodal tDCS applied on the left primary motor cortex would improve participants’ performance and cathodal tDCS would suppress the ability necessary for the tasks, while there would be no significant change in performance for those in the sham condition. (2) There would be no significant change in scores on performance intelligence tasks under any stimulation condition.

## Materials and Methods

### Participants

Sixty-one undergraduate students (45 women; mean age = 20.82 years, *SD* = 1.79) from Renmin University of China participated in the study and were paid for their participation. Participants were randomly assigned into the anodal stimulation group (15 women; *n* = 20), the cathodal stimulation group (13 women; *n* = 20), or the sham stimulation group (17 women; *n* = 21). An sensitivity power analysis was conducted using G*power version 3.1.9.7 (Faul et al., 2007) with the given sample size, an alpha of 0.05, and statistical power of 0.95, revealing that the smallest effect size could be detected in present design was *f* = 0.26, which achieving a medium effect size (0.25 < *f* < 0.4). All participants were right-handed with normal intellectual development, reported no history of neurological or psychiatric diseases, and had not previously participated in a similar intelligence test. Written informed consent was obtained from all participants. The study was approved by the Institutional Review Board of the Department of Psychology at Renmin University of China.

### Experimental Materials

The WAIS-RC consists of 11 subtests, six of which are used for assessing VIQ (Information, Comprehension, Arithmetic, Similarities, Digit Span, and Vocabulary subtests), and the remainder are used for assessing PIQ (Picture Completion, Picture Arrangement, Block Design, Object Assembly, and Digit Symbol) (Gong, 1983). Due to the limitations of the duration of the tDCS effect, only two verbal subtests, Comprehension and Similarities, and two performance subtests, Digit Symbol and Block Design, were selected for intelligence evaluation in the present study. Comprehension subtest evaluates the ability of verbal reasoning by asking participants to explain the meaning of the given words. Similarities subtest is conducted by showing participants with pairs of nouns and asking for answers about in what way they are alike, which reflects the process of conceptualization. In Digit Symbol subtest, participants need to transform the digits into corresponding symbols in 90 s, and scores represent the attentional flexibility. Block Design subtest is regarded as a good assessment of visuospatial ability and participants are required to build blocks into given shapes. The choice of subtests was also based on a previous study, in which researchers found that, out of the six subtests of verbal intelligence, only the scores of Comprehension and Similarities were significantly correlated with the increase in gray matter density in the left primary motor cortex, both with high correlation coefficients, while Digit Symbol and Block Design showed no link to changes in gray matter density in this region, with the smallest coefficients (Ramsden et al., 2011). In addition, cognitive assessment of epilepsy patients who underwent frontal lobe resection in the premotor or supplementary cortex showed a decline in verbal reasoning ability as measured by the Comprehension subtest (Ljunggren et al., 2015).

We restricted the items of each subtest to obtain two experimental materials, A and B, for pre- and post-tDCS measurements. In the WAIS-RC, the items of the Comprehension, Similarities, and Block Design subtests increase in difficulty with the item number. Therefore, the three subtests were divided into two parts based on the parity of the item numbers (e.g., 1, 3, 5, 7, and 2, 4, 6, 8), yielding two experimental materials, A and B. For the Digit Symbol subtest, to avoid memory effects, we randomly re-matched the correspondence between numbers and symbols by computer to form a new set of number-symbols table, which, together with the original table, yielded two experimental materials, A and B, for pre- and post-tests.

Another group of 18 young adults (12 females; mean age = 22.78 ± 2.42 years) were recruited to participate in a pilot study to examine whether individuals’ performance would change with the number of tests, that is, whether a practice effect occurred. Participants first completed Test A and then Test B (or vice versa) after a 20-min break. The order of Test A and Test B was counterbalanced between participants. For the Comprehension, Digit Symbol, and Block Design subtests, the total item scores were analyzed, while for the Similarities subtest, the average score was taken as the subtest score for analysis because of different numbers of items between Test A and Test B. Paired-sample *t*-tests revealed that there were no significant differences of Comprehension, Similarities, and Block Design subtest scores in the post-test relative to the pre-test, but the Digit Symbol scores in the post-test were significantly higher than those in the pre-test, suggesting a potential practice effect in this subtest (see Table 1).

**TABLE 1:** Scores in pre- and post-tests in pilot study (*M ± SD*).

Assessment		Pre-test	Post-test	*t*_(17)_	*p*
Verbal subtests	Comprehension	9.28 ± 1.53	9.50 ± 1.25	–0.75	0.47
	Similarities	0.84 ± 0.10	0.85 ± 0.10	–0.35	0.73
Performance subtests	Digit symbol	72.50 ± 8.45	74.72 ± 8.05	–2.91	**0.01**
	Block design	22.33 ± 1.94	21.83 ± 2.38	1.31	0.21

*Significant differences (p < 0.05) are highlighted in bold.*

### Transcranial Direct Current Stimulation Parameters

**FIGURE 1:**
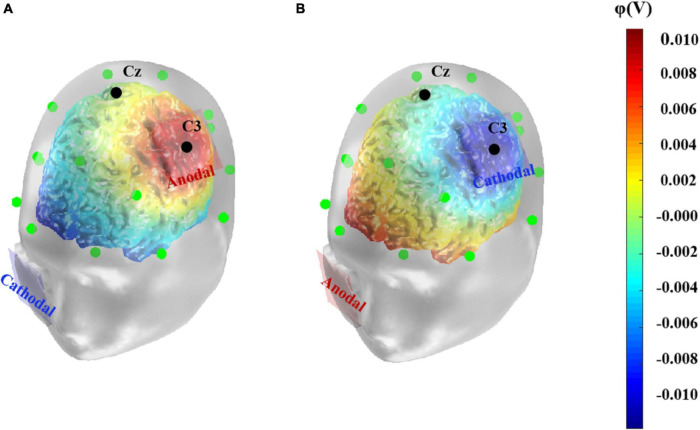
Computational model of anodal **(A)** and cathodal **(B)** tDCS-induced electrical potential. A simulation of the electrical potential induced by tDCS over the left primary motor cortex was computed using COMETS2. The anode or cathode (5 cm× 5 cm) was placed over the left primary motor cortex, corresponding to C3 electrode according to the 10–20 EEG system. The colors denote the simulated electrical potential.

The tDCS was delivered by the DC-STIMULATOR (NeuroConn, Germany) through a pair of saline-soaked sponge electrodes (both 5 cm × 5 cm). To stimulate the left primary motor cortex, the anodal or cathodal electrode was placed over C3 according to the international 10–20 system for EEG electrode placement (Vitali et al., 2002). This location covered the left primary motor cortex coordinates [–47, –9, +30] reported in previous functional magnetic resonance imaging research (Ramsden et al., 2011). The reference electrode was placed on the right check. Previous studies have shown that the stimulation duration is usually set to 5–30 min (Palm et al., 2016), while the current intensity is controlled to 2 mA or less. For the sake of safety and activation effectiveness, in the present study, we conducted constant direct current of 1.5 mA for 20 min in the anodal and cathodal conditions, with a 15 s fade in and fade out time, while the stimulation ramped down to zero after the first 15 s in the sham condition. Participants were seated in a comfortable chair and were asked to relax during the stimulation. Given that the effects of tDCS targeting a specific area may spread to other cortical structures (Fregni and Pascual-Leone, 2007), simulations of tDCS-induced current density, electric field strength or electric potential help to visualize the possible effects of tDCS and to determine the optimal stimulation location (Mendonca et al., 2011). Thus, referring to previous studies (Adelhöfer et al., 2019; Bhattacharjee et al., 2019), we simulated the anodal and cathodal tDCS-induced electrical potentials using the COMETS2 toolbox in MATLAB (Figure 1, Lee et al., 2017).

### Experimental Procedures

The participants were randomly assigned into three groups, who received anodal, cathodal, and sham stimulation, respectively. After entering the laboratory, participants were required to complete one of two tests (Tests A and B), after which they received different types of tDCS for 20 min. Following the stimulations, participants were asked to complete the remaining set of tests immediately. The order of test A and B was counterbalanced between participants. Notably, here we used a sham-controlled, double-blind design in which neither the participants nor the experimenter knew which tDCS the participant was receiving during the experiment, and for the participants in the sham group, the sensation of mild irritation in the scalp elicited during the initial phase of the experiment reassured them that they were receiving a real stimulus, which could help to control for possible experimenter effects as well as placebo effects. Tests A and B were completed in 20 min each, and the entire experiment took about 1 h.

### Data Analysis

Scores of each subtest were analyzed using a mixed two-way repeated-measures analysis of variance (ANOVA) with one between-subjects factor (tDCS group: anodal, cathodal, and sham) and one within-subject factor (test order: pre-test and post-test). The Shapiro-Wilk test revealed that the assumptions of normality were violated in some conditions of the data. Because of the relatively high tolerance of repeated measures ANOVAs for non-normal data (Vasey and Thayer, 1987; Berkovits et al., 2000; Schmider et al., 2010; Blanca et al., 2018), and given that the parametric analysis is a more robust method compared to the non-parametric analysis under the general presupposition that the population intelligence is normally distributed (Burt, 1957; Glass et al., 1972; Plomin and Deary, 2015), we continued to report parametric statistics below. The results of Shapiro-Wilk test and non-parametric analysis are provided in Supplementary Information. None of our main results were affected by the change in analysis methods. The post hoc comparison was performed with Bonferroni correction to examine the main effects of each factor and pairwise comparison analysis was used to explore the interaction effects. Partial eta-squared (η*_*p*_*^2^) was computed as an index of effect size in the ANOVA models, with 0.05 representing a small effect, 0.1 representing a medium effect, and 0.2 representing a large effect (Cohen, 1973). All statistical analyses were performed in SPSS 24.0 (IBM Inc.).

## Results

**FIGURE 2:**
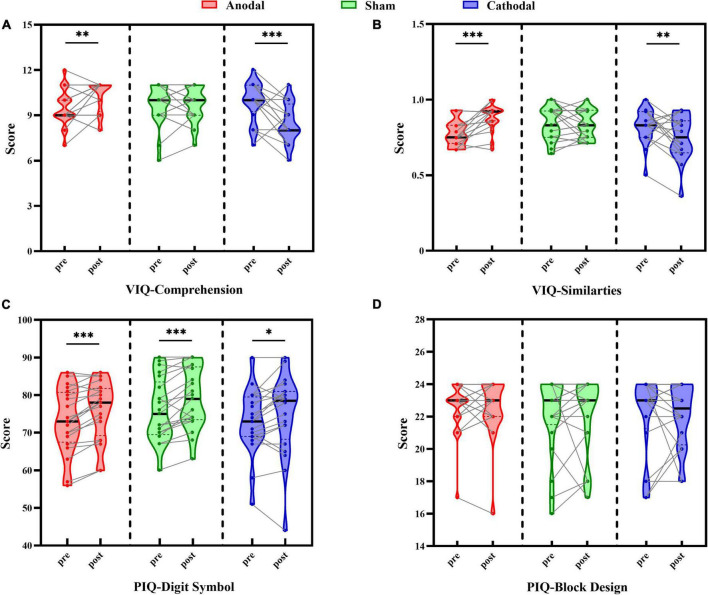
Comparison between pre-test and post-test scores under each tDCS condition (anodal, cathodal, and sham) in Comprehension subtest **(A)**, Similarities subtest **(B)**, Digit Symbol subtest **(C)**, and Block Design subtest **(D)**. Individual participant data points, densities, medians, and quartiles are displayed, *p < 0.05, ^**^p < 0.01, ^***^p < 0.001.

Pre- and post-test scores in each stimulus condition are shown in Figure 2. Scores of the Comprehension and Similarity subtests as two verbal intelligence subtests were each analyzed with the two-way repeated-measures ANOVA. For the Comprehension subtest scores, the results showed no significant main effects of test order, [*F*(1, 58) = 1.73, *p* = 0.194, η*_*p*_*^2^ = 0.03], and stimulus condition, [*F*(2, 58) = 2.65, *p* = 0.079, η*_*p*_*^2^ = 0.08], although there was a significant interaction between test order and stimulus condition, [*F*(2, 58) = 15.71, *p* < 0.001, η*_*p*_*^2^ = 0.35] (Figure 2A). Further pairwise comparison analyses revealed a significant increase in post-test scores compared to pre-test scores for the anodal group, *t*(19) = 2.79, *p* = 0.012, Cohen’s *d* = 0.63, 95% CI = [0.20, 1.40], a significant decrease in the cathodal group, *t*(19) = –4.31, *p* < 0.001, Cohen’s *d* = 0.96, 95% CI = [–2.15, –0.75], and no difference between pre- and post-test scores for the sham group, *t*(20) = 0.00, *p* = 1.000, Cohen’s *d* = 0.00, 95% CI = [–0.48, 0.48].

The same pattern of results was found in the Similarities subtest scores (Figure 2B), with no main effects of test order, [*F*(1, 58) = 0.28, *p* = 0.598, η*_*p*_*^2^ = 0.01], or stimulus condition, [*F*(2, 58) = 1.83, *p* = 0.170, η*_*p*_*^2^ = 0.06], although there was a significant interaction between test order and stimulus condition, [*F*(2, 58) = 12.52, *p* < 0.001, η*_*p*_*^2^ = 0.30]. Pairwise comparison analysis indicated that the score of the post-test compared to the pre-test was reliably improved in the anodal group, *t*(19) = 4.54, *p <* 0.001, Cohen’s *d* = 1.01, 95% CI = [0.06, 0.15], and significantly decreased in the cathodal group, *t*(19) = –2.42, *p* = 0.026, Cohen’s *d* = 0.54, 95% CI = [–0.14, –0.01], while no change was found in the sham group, *t*(20) = –0.18, *p* = 0.856, Cohen’s *d* = 0.04, 95% CI = [–0.05, 0.04]. These results suggested that the changes between the verbal intelligence subtests were moderated by the type of tDCS, reflecting the beneficial effect of anodal stimulation on performance and the negative influence on scores of cathodal stimulation, while sham stimulation showed no evident effect on the scores.

Scores of the Digit Symbol and Block Design subtests, as two performance intelligence subtests, were each analyzed with two-way repeated-measures ANOVA. For the Digit Symbol subtest scores (Figure 2C), the results revealed a significant main effect of test order, [*F*(1, 58) = 36.81, *p* < 0.001, η*_*p*_*^2^ = 0.39], but not of stimulus condition, [*F*(2, 58) = 1.07, *p* = 0.348, η*_*p*_*^2^ = 0.04], nor an interaction between test order and stimulus type, [*F*(2, 58) = 0.72, *p* = 0.492, η*_*p*_*^2^ = 0.02]. Participants’ scores on the post-test (76.63 ± 1.15) were significantly better than those on the pre-test (73.95 ± 1.09), suggesting a stable improvement by practice effect that was independent of the stimulus condition.

With respect to the Block Design subtest scores (Figure 2D), there were no significant main effects of test order, [*F*(1, 58) = 0.04, *p* = 0.840, η*_*p*_*^2^ = 0.001], or stimulus condition, [*F*(2, 58) = 0.29, *p* = 0.749, η*_*p*_*^2^ = 0.01], and there was no interaction between test order and stimulus type, [*F*(2, 58) = 0.06, *p* = 0.945, η*_*p*_*^2^ = 0.002], indicating that there was no difference in participants’ performance before and after each type of stimulus. These findings demonstrated that stimulus condition had no effect on the scores of performance intelligence subtests, but there was a possible practice effect in the Digit Symbol subtest that reflected general improved performance in the post-test relative to the pre-test, which was also consistent with the results in our pilot study.

## Discussion

In the present study, we utilized tDCS to examine the influence of cortical excitability of the left motor area on performance in the WAIS-RC. The results showed that in the verbal intelligence tests, for both the Comprehension and Similarities subtests, participants obtained significantly increased scores under anodal stimulation and decreased scores under cathodal stimulation, while no obvious change of scores was found in the sham condition. Such modulating effects were not observed in the performance intelligence tests in which tDCS showed no influence on the task scores, indicating that tDCS applied on the left primary motor cortex had a specific effect on individuals’ performance on verbal but not performance intelligence.

The present findings are consistent with the association between the left primary motor cortex and performance in verbal tasks found in previous studies. Ramsden et al. (2011) reported a positive correlation between the development of verbal intelligence and changes in gray matter structure (both volume and density) in the left motor area in adolescence, and another study found that epilepsy patients who underwent surgery on the premotor or supplementary motor area showed impaired verbal reasoning ability (assessed by the Comprehension subtest) (Ljunggren et al., 2015), suggesting that the left motor cortex may be a critical area for the maturation of verbal intelligence, and its injury is associated with marked deficits in this ability, indicating an association between this area and verbal intelligence.

However, so far, relatively few studies have addressed the exact level at which the left motor area influences verbal intelligence; that is, which abilities or processes reflected by verbal intelligence are correlated with this region? Existing evidence has mostly focused on its possible effect on speech production and concept comprehension, both of which are processes essential for the completion of a verbal intelligence task. Fluent articulation may be a fundamental process not only for orally intellectual test, but also for other verbal tasks. It has been supported that the primary motor cortex involves an overt articulatory process (Huang et al., 2002; Correia et al., 2020). Another thread of evidence links the left primary motor area to concept comprehension, which is particularly relevant to the verbal subtests we selected. Various studies have suggested that activation of this region has effects on semantic word generation, picture naming, novel lexicon learning and retrieval, and word-to-picture matching tasks (Liuzzi et al., 2010; Meinzer et al., 2014, 2016; Schomers et al., 2015), all of which show deployment of conceptual knowledge. It is believed that such conceptual knowledge depends on a widely distributed neural network, including a broad range of modality-specific and shared-amodal areas (Patterson et al., 2007). With persistent reinforcement of synaptic circuits of regions and connections therein, the knowledge of the facts and concepts are better reserved (Wiltgen et al., 2004). Therefore, in this view, changes in gray matter at the left primary motor cortex found in adolescence may reflect the accumulation of semantic concepts, which is crucial for verbal tasks. Meanwhile, increased gray matter may be accompanied by increased functional activation (Josse et al., 2009). It could be reasoned that excitability of the left primary motor cortex may influence conceptualization of the given items, and further affects individuals’ performance in verbal intelligence tasks, as shown in our study.

It has been found that tDCS applied on the left motor cortex could affect cortical excitability in adjacent regions and alters functional cortical network connectivity (Antal et al., 2011; Polanía et al., 2011). Wang et al. (2019) found that anodal tDCS administered on the left primary motor cortex promoted speech function in patients with post-stroke aphasia, and the EEG approximate entropy indices showed that not only the targeted site but also the dorsolateral prefrontal cortex and Broca’s area in the ipsilateral hemisphere were significantly activated. This indicated improved connectivity of speech-related areas in the articulation network. Consistent with these findings, in the current study, simulation of electrical potential showed increased levels of potentials in Broca’s area and parts of the left superior temporal gyrus and middle temporal gyrus compared to other cortical areas. Broca’s area is closely related to speech processing and verbal working memory (Chein et al., 2002), and it is reported that gray matter density in this area had a positive correlation with verbal intelligence in young adults (Konrad et al., 2012), while the temporal cortex is considered the key area for storage of semantic knowledge. In addition, patients with mesial temporal lobe epilepsy exhibit significant impairments in verbal intelligence (Kim et al., 2003). Thus, anodal tDCS conducted on the left primary motor cortex may not only affect performance in tests directly, by modulating the excitability of this site, but also indirectly influence performance by promoting the activation of other regions correlated with verbal tasks. More empirical evidence are necessary for supporting this hypothesis. In addition to the simulation of electrical potential induced by tDCS, other dynamic measures (e.g., the approximate entropy and ERP components) may provide a more direct depiction of the cortical activation during the tasks (Yuan et al., 2017; Nikolin et al., 2018; Zhang et al., 2020).

In contrast to the results observed in the verbal intelligence subtests, the findings of performance intelligence subtests showed that scores did not change under any type of stimulation, suggesting that the degree of excitation in the left primary motor cortex had no effect on the ability corresponding to fluid intelligence. However, there was a practice effect in the Digit Symbol subtest, shown in both the pilot study and the formal experiment, with participants’ post-test scores significantly better than their pre-test scores for each stimulation type. The Digit Symbol subtest asks participants to match symbols with their corresponding numbers in a limited time, and it is widely use to evaluate learning ability, visual-motor coordination, and processing speed (Murstein and Leipold, 1961; Kreiner and Ryan, 2001). Although we formed two set of tests with different number-symbol correspondence, it could be assumed that after the pre-test, participants had become familiar with the task requirements, and thus became more proficient at performing the task in the post-test. Previous research also found that repeated exposure to Digit Symbol tasks could improve performance across multiple task sessions in healthy individuals in good condition (Van Dongen et al., 2003). This result also appears steadily in variant versions of the task (Honn et al., 2020). In the Block Design subtest, there was no change in pre- and post-test scores, which may have been due to a larger difference between items and the higher demands of spatial reasoning ability, which could have made it more difficult to elicit a rapid practice effect compared to the Digit Symbol subtest.

Several limitations to this work should be noted. First, because of the limited duration of tDCS effects, we only selected two WAIS-RC subtests for the assessment of verbal intelligence in the present study, which are not sufficient to cover all the abilities corresponding to this kind of intelligence. Verbal intelligence is believed to reflect the breadth and depth of acquired knowledge and processing abilities that are important to one’s culture, which includes factual information, comprehension, concepts, rules, relationships, and the procedures of utilizing learned skills (Flanagan and Dixon, 2013). The Comprehension subtest mainly evaluates individuals’ ability to make moral judgments and their understanding of these phenomena in social interaction (e.g., participants are asked to explain the meaning of *marriage*), while the Similarities subtest focuses on the ability to generalize the common properties of different semantic concepts (e.g., participants are asked to say in what way a *poem* and a *statue* are similar) (Fernaeus and Hellstrom, 2018). Although partly representing the ability corresponding to verbal intelligence, performance in these two subtests still cannot provide a complete picture of this kind of intelligence. Further studies are needed to investigate whether findings in the present study could be replicated in other tests related to verbal intelligence, or whether the beneficial effect of the anodal tDCS on verbal performance is task-dependent.

Second, the results of the present study only suggest that the cortical excitability of the left primary motor cortex influenced performance in verbal intelligence tests, but cannot provide insights into the underlying mechanism. The individual’s performance in verbal intelligence tests depends on both static declarative knowledge and the ability to dynamically integrate acquired knowledge to form an appropriate answer to a given question (Flanagan and Dixon, 2013). It is thus a complex process that involves semantic comprehension, phonological representation, and even advanced executive functions so that a precise response can be formed (Lee et al., 2014). Projecting the mental process to its neural basis, evidence from some neuroimaging studies has indicated that structural changes in the left motor area may correlate with the accumulation of concept knowledge or sensorimotor skills (Patterson et al., 2007; Ramsden et al., 2011), which could contribute to richer reserves of semantic knowledge or more fluent articulation, respectively. Furthermore, it was found that tDCS applied on the left motor area could extensively enhance the cortical excitability of other adjacent regions in articulatory network (Wang et al., 2019). In the present study, simulated electrical potentials induced by tDCS in the Broca’ s area and left temporal area showed similar patterns, which might explain the enhanced performance in such tasks. Given all these possibilities, which task-related processes were specifically influenced beyond the overall positive effect caused by anodal tDCS? Are other verbal-related brain regions actually affected by tDCS and contributing to the performance promotion? Future work on this topic should seek to further explore these uncovered questions.

Finally, although the current study was conducted in a randomized, double-blinded way with a sham group recruited to mitigate the potential placebo effect, which has been widely implemented in previous tDCS studies (Giustolisi et al., 2018; Rivera-Urbina et al., 2019; Mondino et al., 2020), it may still be insufficient to rule out all of confounding factors, as sham treatment may inevitably induce sensation different from active conditions (Fietsam et al., 2021) and cause some mild physiological effects that are easily overlooked (Nikolin et al., 2018). Thus, using multi-electrode montages and adding an active control group targeting the brain area that irrelevant to certain tasks may be optional ways to address this issue in the future (Fonteneau et al., 2019).

In sum, the present study examined the effects of excitability in the left primary motor cortex on verbal and performance intelligence by utilizing the tDCS technique. The results showed that even a single session of anodal stimulus applied on the left primary motor cortex could promote performance on verbal intelligence tests, while a cathodal stimulus had a negative effect on performance in this intelligence dimension. In addition, different directions of tDCS administered on the same brain region had no effect on scores on performance intelligence tests. The present study has showed a direct relationship between the excitability of the left primary motor cortex and performance in verbal intelligence tasks. However, the underlying mechanisms and exact role of this brain area in this process remain to be further explored. The unveiling of these questions will provide more evidence for the causal influence of the left primary motor area on verbal intelligence.

## Data Availability Statement

The raw data supporting the conclusions of this article will be made available by the authors, without undue reservation.

## Ethics Statement

The studies involving human participants were reviewed and approved by Institutional Review Board of the Department of Psychology at Renmin University of China. The participants provided their written informed consent to participate in this study.

## Author Contributions

YH: literature synthesis, data analysis, data interpretation, manuscript drafting, writing and editing, and creation of figures and tables. YLZ: data interpretation, manuscript writing and editing. YZZ: methodology, data curation, and data analysis. XM: conceptualization, data interpretation, project administration, supervision, manuscript writing, and editing. All authors contributed to the article and approved the submitted version.

## Conflict of Interest

The authors declare that the research was conducted in the absence of any commercial or financial relationships that could be construed as a potential conflict of interest.

## Publisher’s Note

All claims expressed in this article are solely those of the authors and do not necessarily represent those of their affiliated organizations, or those of the publisher, the editors and the reviewers. Any product that may be evaluated in this article, or claim that may be made by its manufacturer, is not guaranteed or endorsed by the publisher.
