# Supplementary material for: Effects of Transcranial Direct Current Stimulation Over the Left Primary Motor Cortex on Verbal Intelligence
Source: Front Hum Neurosci. 2022 May 26;16:888590. doi: 10.3389/fnhum.2022.888590 (PMC9177941; doi:10.3389/fnhum.2022.888590)
Supplement: Supplementary file 1 [file Data_Sheet_1.docx]

**Supplementary information**

*The Shapiro-Wilk Test*

Normality assumptions were tested using Shapiro-Wilk test, which is appropriate for small samples. As shown in Table S1, the scores in some conditions violated the normality assumption.

.

**Table S1.** The Shapiro-Wilk normality test.

| **Assessment** | | **order** | **group** | **Skewness**  ***M*(*SE*)** | **Kurtosis**  ***M*(*SE*)** | ***df*** | ***p*** |
| --- | --- | --- | --- | --- | --- | --- | --- |
| Verbal  subtests | Comprehension | pre | Anodal | 0.104(0.512) | 0.190(0.992) | 20 | 0.221 |
|  |  |  | Sham | −1.480(0.501) | 2.521(0.972) | 21 | **0.001** |
|  |  |  | Cathodal | −0.768(0.512) | 0.038(0.992) | 20 | **0.019** |
|  |  | post | Anodal | −1.228(0.512) | 0.042(0.992) | 20 | **0.000** |
|  |  |  | Sham | −0.696(0.501) | −0.017(0.972) | 21 | **0.018** |
|  |  |  | Cathodal | 0.208(0.512) | −0.401(0.992) | 20 | 0.161 |
|  | Similarities | pre | Anodal | 0.452(0.512) | 0.716(0.992) | 20 | 0.212 |
|  |  |  | Sham | −0.193(0.501) | −0.931(0.972) | 21 | **0.040** |
|  |  |  | Cathodal | −1.226(0.512) | 2.675(0.992) | 20 | **0.035** |
|  |  | post | Anodal | −1.131(0.512) | 0.749(0.992) | 20 | **0.025** |
|  |  |  | Sham | 0.043(0.501) | −1.353(0.972) | 21 | **0.013** |
|  |  |  | Cathodal | −0.836(0.512) | 1.109(0.992) | 20 | 0.097 |
| Performance  subtests | Digit Symbol | pre | Anodal | −0.409(0.512) | −0.460(0.992) | 20 | 0.377 |
|  |  |  | Sham | 0.204(0.501) | −0.815(0.972) | 21 | 0.547 |
|  |  |  | Cathodal | −0.604(0.512) | 1.526(0.992) | 20 | 0.402 |
|  |  | post | Anodal | −0.789(0.512) | −0.223(0.992) | 20 | 0.262 |
|  |  |  | Sham | −0.135(0.501) | −0.882(0.972) | 21 | 0.078 |
|  |  |  | Cathodal | −1.223(0.512) | 2.217(0.992) | 20 | 0.075 |
|  | Block Design | pre | Anodal | −2.191(0.512) | 6.469(0.992) | 20 | **0.001** |
|  |  |  | Sham | −1.438(0.501) | 1.294(0.972) | 21 | **0.000** |
|  |  |  | Cathodal | −1.331(0.512) | 0.248(0.992) | 20 | **0.000** |
|  |  | post | Anodal | −2.384(0.512) | 7.764(0.992) | 20 | **0.000** |
|  |  |  | Sham | −1.230(0.501) | 0.084(0.972) | 21 | **0.000** |
|  |  |  | Cathodal | −0.870(0.512) | −0.196(0.992) | 20 | **0.009** |

Note: Significant differences (*p* < 0.05) are highlighted in bold.

*Non-parametric Analysis*

To examine the pre- and post-test differences in the four subtests (Comprehension, Similarities, Digit Symbol and Block Design) for the three groups (anodal, sham, and cathodal), pair-wise Wilcoxon signed-rank tests were conducted for each condition separately. Descriptive statistics and test results are shown in Table S2.

For the verbal intelligence subtests, the anodal group showed significant post-test increases in both Comprehension and Similarities subtest scores, while the cathodal group showed decreases in both, and there were no significant changes in the sham group, revealing the same pattern as found in parametric statistics.

For the Digit Symbol subtest of performance intelligence, similar practice effects were observed in the anodal and sham groups, i.e., higher test scores on the post-test, while no significant difference were found between the pre-test and post-test scores for the cathodal group, although the post-test scores remained higher, which was the only difference from the parametric statistical results. In the Block Design subtest, no reliable differences were found between the pre-test and post-test scores in either tDCS group.

Overall, the statistical results of the nonparametric analysis are consistent with the main results of the parametric analysis, which further confirms the current findings.

**Table S2.** Non-parametric analysis for scores under different conditions.

| **Assessment** | **group** | **Pre-test** | **Post-test** | **Z** | ***p*** |
| --- | --- | --- | --- | --- | --- |
| Comprehension | Anodal | 9.00 (9.00~10.00) | 11.00 (9.25~11.00) | −2.45 | **0.014** |
|  | Sham | 10.00 (9.00~11.00) | 10.00 (9.00~11.00) | 0.00 | 1.000 |
|  | Cathodal | 10.00 (9.25~11.00) | 8.00 (8.00~9.75) | −2.99 | **0.003** |
| Similarity | Anodal | 0.75 (0.71~0.83) | 0.92 (0.86~0.93) | −3.40 | **0.001** |
|  | Sham | 0.83 (0.75~0.93) | 0.83 (0.75~0.93) | −0.37 | 0.713 |
|  | Cathodal | 0.83 (0.75~0.92) | 0.75 (0.65~0.86) | −2.17 | **0.030** |
| Digit Symbol | Anodal | 73.00 (67.50~80.75) | 78.00 (69.25~81.75) | −3.51 | **0.000** |
|  | Sham | 75.00 (69.50~83.50) | 79.00 (73.50~87.50) | −3.33 | **0.001** |
|  | Cathodal | 73.00 (69.00~79.50) | 78.50 (68.25~81.00) | −1.62 | 0.106 |
| Block Design | Anodal | 23.00 (22.00~23.00) | 23.00 (22.00~24.00) | −0.15 | 0.883 |
|  | Sham | 23.00 (21.50~24.00) | 23.00 (21.00~24.00) | −0.96 | 0.336 |
|  | Cathodal | 23.00 (21.25~24.00) | 22.50 (20.25~24.00) | −0.11 | 0.915 |

Note: Significant differences (*p* < 0.05) are highlighted in bold.
